# A Case of Hydrophobia in the South of France

**Published:** 1852-07

**Authors:** 


*A Case of Hydrophobia in the South of France.*—M. Guyon, of Valence, in the South of France, has communicated to the Société d'Emulation of Paris, the report of a case of hydrophobia, drawn up in a very graphic manner, and presenting some points of interest. The patient was a nailer, about thirty years of age, who was bitten by a little dog, which he had never seen before, and which was quarrelling with his own. No signs of disease were noticed in the strange dog; it was seen for several hours afterwards to play about, gnaw bones, &c., and was subsequently lost sight of.

The medical man who was called in wished to cauterize the three small wounds with the hot iron, but the patient refused, and his surgeon readily yielded, as the dog was supposed to be healthy. Forty days (the period vulgarly believed necessary for incubation) passed away, and the patient now exclaimed that he had been more afraid than hurt, for he had felt uneasy in his mind ever since the accident. About a fortnight after this, the man began to give signs of peevishness, and became quarrelsome and abusive. He soon complained of pain in the vicinity of the pharynx, difficulty of swallowing came on, and the fits of dyspnœa, when water was placed in his mouth, were dreadful.

The surgeon in attendance noticed that the papillæ at the base of the tongue were much enlarged, that the epiglottis became quite erect, and resembled in shape and color a small cherry. Every time that he succeeded in lowering it, the respiration became easier. There was no horror of water, but a difficulty of swallowing it, the fauces, larynx and pharynx being highly injected. The spasmodic fits succeeded each other with fearful rapidity, the cellular tissue of the neck and chest became emphysematous, and the patient died asphyxiated the day after the attack.

No post-mortem examination was allowed, but the larynx and posterior portion of the tongue were taken out; they did not present any distinct alterations. M. Guyon appends to the case the following remarks:

1st. It is clear, judging from the case just related, that a dog can communicate the disease whilst the latter is yet latent; and if it be the salivary secretion which conveys the virus, it does not seem necessary that the animal have a great abundance of it, and foam at the mouth, for the fluid to be contagious as is vulgarly supposed. 2nd. It has been imagined that fear and apprehension have much influence on the development of hydrophobia; but this case shows that the disease may manifest itself more than a fortnight *after* every sort of apprehension has passed away. 3rd. The peculiar rising and stiffening of the epiglottis noticed in this case, has not been observed before, and would sufficiently explain the laryngeal spasms, which eventually brought on asphyxia. The phenomena of hydrophobia might thus be accounted for, without having recourse to a lesion of the nervous centres, which lesion has, in fact, never been demonstrated by autopsy. 4th. The emphysema of the neck and chest was probably due to the rupture of some pulmonary vesicles during the violent spasms which the patient had experienced. This circumstance shows very clearly that there must be direct communications between the general subcutaneous cellular tissue and the submucous areolar texture of the lungs. 5th. The hypertrophied papillæ at the base of the tongue, which were seen very early in the course of the disease, would explain the sense of pharyngeal constriction of which the patient complained at the very outset. Thus it would finally appear, that it is principally in the pharynx and larynx that hydrophobia becomes localised, although it is likely that the disease arises from a virulent infection of the whole organism.—*Ibid.*

